# Comparing autologous blood, corticosteroid, and a combined injection of both for treating lateral epicondylitis: a randomized clinical trial

**DOI:** 10.1186/s10195-024-00772-4

**Published:** 2024-07-04

**Authors:** Albert Cakar, Ozgur Dogus Gozlu

**Affiliations:** Department of Orthopedics and Traumatology, Istanbul Training and Research Hospital, Istanbul, Turkey

**Keywords:** Lateral epicondylitis, Tennis elbow, Autologous blood, Corticosteroid

## Abstract

**Background:**

Because lateral epicondylitis is a common musculoskeletal disorder that affects the forearm's extensor tendons, an effective therapeutic approach should reverse the degeneration and promote regeneration. This study aimed to compare the efficacies of autologous blood (AB) injection, corticosteroid (CS) injection, and a combined injection of both in treating lateral epicondylitis (LE), hypothesizing that the combined approach might offer immediate symptom resolution and a lower recurrence.

**Materials and methods:**

A total of 120 patients diagnosed with lateral epicondylitis were systematically distributed among three distinct therapeutic injection groups. Those in the AB group were administered 1 ml of autologous venous blood mixed with 2 ml of 2% prilocaine HCl. Participants in the CS category were given 1 ml of 40 mg methylprednisolone acetate mixed with 2 ml of 2% prilocaine HCl. Meanwhile, patients in the combined group received a mixture containing 1 ml each of autologous venous blood and 40 mg methylprednisolone acetate along with 1 ml of 2% prilocaine HCl. Prior to receiving their respective injections, a comprehensive assessment of all participants was carried out. Follow-up assessments were subsequently conducted on days 15, 30, and 90 utilizing metrics of the patient-rated tennis elbow evaluation (PRTEE) and measurements of hand grip strength (HGS).

**Results:**

One patient dropped out from the combined group, and 119 patients completed the trial. No complications were recorded during the course of follow-up. By day 15, all groups had demonstrated significant PRTEE improvement, with CS showing the most pronounced reduction (*p* = 0.001). However, the benefits of CS had deteriorated by day 30 and had deteriorated further by day 90. The AB and AB + CS groups demonstrated sustained improvement, with AB + CS revealing the most effective treatment, achieving a clinically significant improvement in 97.4% of the patients. The improved HGS parallelled the functional enhancements, as it was more substantial in the AB and AB + CS groups (*p* = 0.001), corroborating the sustained benefits of these treatments.

**Conclusions:**

The study concluded that while AB and CS individually offer distinct benefits, a combined AB + CS approach optimizes therapeutic outcomes, providing swift and sustained functional improvement with a lower recurrence rate. These findings have substantial clinical implications, suggesting a balanced, multimodal treatment strategy for enhanced patient recovery in LE.

*Level of evidence*: Randomized clinical trial, level 1 evidence.

*Trial registration*: NCT06236178.

## Introduction

Lateral epicondylitis (LE) is a common musculoskeletal disorder affecting the extensor tendons of the forearm, manifesting as pain on the lateral side of the elbow and functional limitation during daily activities. Despite its name suggesting inflammation, histopathological examinations of the tendon reveal non-inflammatory angiofibroblastic tendinosis accompanied by neovascularization, disorganized collagen, and mucoid degeneration [1, 2]. Since tendon degeneration is the underlying pathology, an effective therapeutic approach should reverse the degeneration to regeneration, ensuring symptom relief without recurrence.

Although many treatments exist, a consensus on the optimal single treatment for LE remains elusive. Traditionally, injections have served as a preliminary measure before resorting to surgical interventions, especially in resistant cases. However, an increasing number of alternative injections have been described in recent literature [3]. Newer injection therapies, such as autologous blood (AB) injections and platelet-rich plasma (PRP), have gained prominence and found favor among orthopedic surgeons. Multiple studies have attested to the safety and efficacy of AB and PRP injections as treatment modalities [4–7]. However, corticosteroid (CS) injections continue to be among the most commonly employed treatments for LE. A significant drawback of CS injections is their high recurrence rate. Patients typically experience a swift recovery immediately after injection, which endures for about a month. Unfortunately, this transient relief—often referred to as the 'honeymoon period'—is short-lived, leading to a recurrence of symptoms. Such a response pattern is well documented in existing studies [8]. Conversely, AB injection therapy tends to proffer a gradual yet consistent reduction in symptoms, boasting a recurrence rate markedly lower than that of CS. It is postulated that AB addresses the intrinsic degeneration of the tendon, with its restorative process inherently spanning a more extended period [9–11].

We hypothesized that a combination of CS and AB injection might offer a superior treatment for LE. This combination may provide a faster resolution of symptoms due to the initial action of CS and provide a lower recurrence rate due to the regenerative action of AB. The purpose of this randomized clinical trial is to test three different types of injections for the treatment of LE: (1) AB, (2) CS, and (3) their combination (AB + CS).

## Materials and methods

### Patients and study design

A randomized clinical trial was carried out on eligible patients with a diagnosis of LE who presented to our outpatient clinic. The diagnosis of LE was made with typical symptoms and physical examination findings, including pain and tenderness localized to the origin of the forearm extensors and discomfort on provoked wrist and middle-finger extension. Patients whose complaints persisted for more than 3 months were evaluated for inclusion in the study.

Patients with a history of recent trauma, congenital or neuromuscular disease, or abnormality of the upper limb, previous upper limb surgery, a history of rheumatic disease, a history of cervical disc pathology or carpal tunnel syndrome, systemic corticosteroid treatment, any previous local injection treatment, and, finally, a history of a previous allergic reaction towards local anesthetics and corticosteroids were all excluded from the study. All patients were over 18 years of age. The local ethical committee approved the study protocol (approval number: 2023/137), and all patients gave their informed consent prior to their inclusion in the study. This prospective study was carried out following the ethical standards laid down in the 1964 Declaration of Helsinki and its later amendments. This research complies with the CONSORT guidelines for conducting randomized controlled trials.

### Treatment groups and randomization

After taking their informed consent, the patients were randomly allocated to three treatment groups (AB versus CS versus their combination) with sealed envelopes prepared by a computer-based random number generator. A total of 120 envelopes were divided into three equal groups, 40 patients in each group. Patients in the AB group received 1 ml of autologous venous blood mixed with 2 ml of 2% prilocaine HCl, patients in the CS group received 1 ml of 40 mg methylprednisolone acetate mixed with 2 ml of 2% prilocaine HCl, and patients in the combined group (AB + CS) received 1 ml of autologous venous blood and 1 ml of 40 mg methylprednisolone acetate mixed with 1 ml of 2% prilocaine HCl. Each group received an equal amount (3 ml) of injected material. Venous blood was collected from the antecubital fossa of the ipsilateral extremity. The senior author performed all injections, and neither the physician nor the patient was blinded to the treatment modality. The injection was administered in aseptic conditions, and the needle was introduced over the point of maximal tenderness; the content of the syringe was injected at once to prevent further bleeding. All patients were instructed to abstain from heavy work, and no additional therapy, including NSAIDs or physiotherapy, was prescribed.

### Follow-up and outcome measures

Functional outcomes were assessed with the patient-rated tennis elbow evaluation questionnaire (PRTEE). This outcome score consisted of 15 items related to three subscales, namely pain (five questions), specific activities (six questions), and daily activities (four questions). The total score ranges between 0 and 100 points; 0 points designates the best functional outcome, whereas 100 points designates the worst functional outcome [12, 13]. To interpret the PRTEE results, a minimum clinically important difference (MCID) value previously described by Poltawiski et al. was used [14]. According to his study, a 37% decrease in PRTEE is considered a complete recovery or a clinically significant change. The PRTEE was assessed before the injection (baseline values) on days 15, 30, and 90 in the same manner by the senior author. The same author performed follow-up and data collection. No patients received more than one injection in this clinical trial.

Hand grip strength (HGS) was measured with a digital hand dynamometer before and after injection in all patients. The senior author performed the measurements according to the American Society of Hand Therapists guidelines [15]. Since HGS varies according to factors such as height, weight, age, and gender, each patient's hand grip strength was calculated as a percentage increase from baseline.

### Sample size calculation

In our pursuit to determine the optimal sample size for our study on LE treatments utilizing PRTEE scores, we based our assumptions on several crucial parameters. We anticipated a 37% change in baseline PRTEE scores and considered a standard deviation (SD) of 10, drawing from previous studies. We adopted a conventional significance level (*α*) of 0.05 and aspired for a power of 0.90, applying the ANOVA testing methodology for three distinct treatment groups. Our computations indicated that to discern this 37% shift in PRTEE scores with the targeted power and significance level, a sample size of roughly 33 participants per group would be essential. Thus, given the three treatment groups in our research design, the aggregate sample size was estimated at 99 participants. However, taking into account a 20% dropout rate, the study included a total of 120 patients, with 40 patients in each group.

### Statistical analysis

Continuous variables were stated as the mean and standard deviation, and categorical variables as the percentage and the frequency distribution. The conformity of the data to a normal distribution was tested using the Kolmogorov–Smirnov test. For the comparison of continuous variables between the three groups, ANOVA was used for the data that fitted the normal distribution, and the independent-sample Kruskal–Wallis test was used for the data that did not fit the normal distribution. Categorical variables were compared using a chi-square test. The Friedman test was used to assess the repeated measurements of the same group. *p* < 0.05 was considered to indicate a statistically significant alpha error.

## Results

**Fig. 1:**
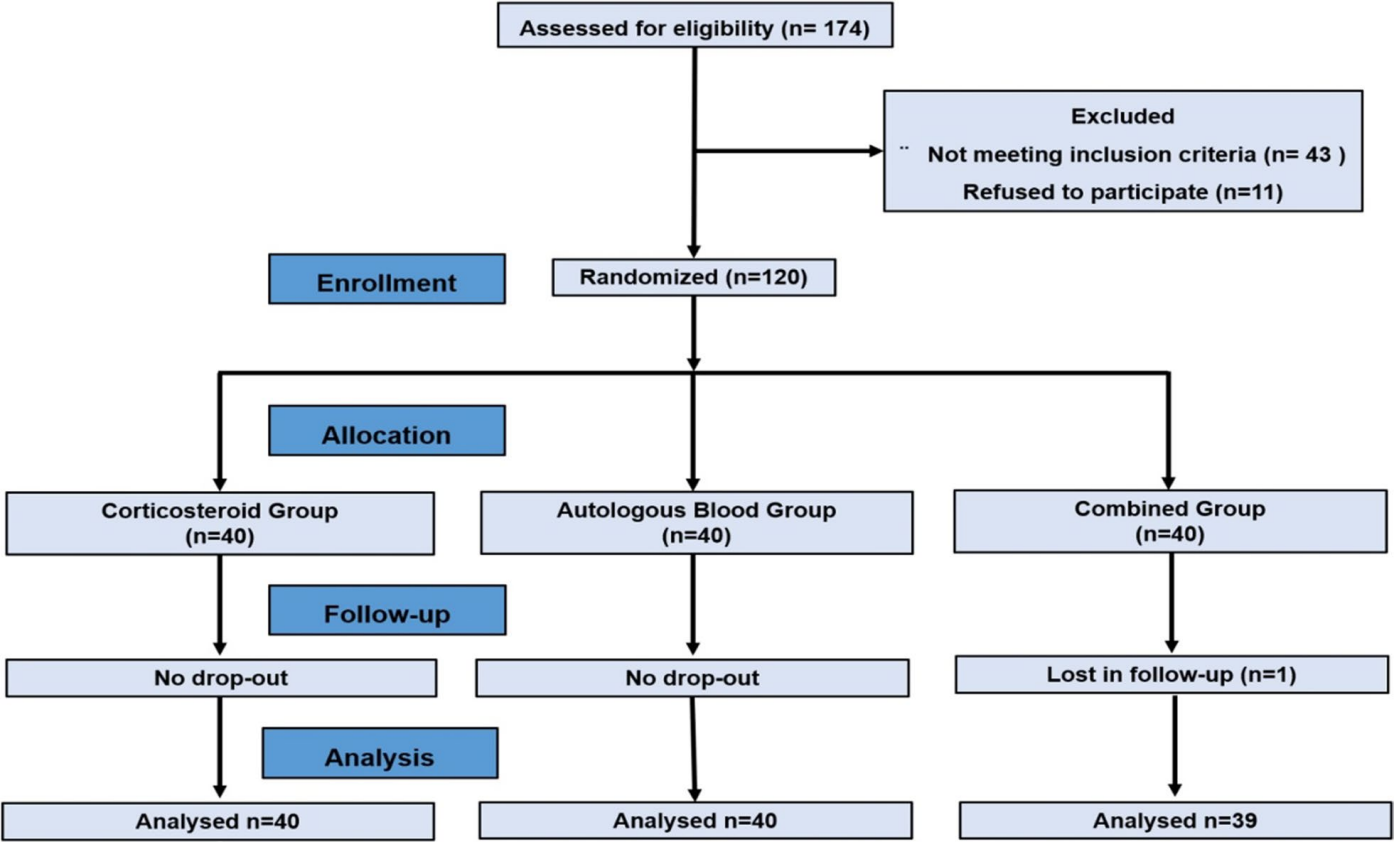
Patient flow chart

One of the patients in the combined injection group was excluded from the study due to non-attendance at the controls. Consequently, 119 patients completed the study, yielding a follow-up rate of 99.1%. The baseline demographic and clinical characteristics of both groups were statistically comparable and are presented in Table 1. During the follow-up period, no complications were observed, including infection, skin atrophy, neurovascular damage, or tendon ruptures. The flow chart is presented in Fig. 1.

**Table 1:** Comparison of baseline demographic and clinical characteristics between treatment groups

Variables	AB group (*n* = 40)	CS group (*n* = 40)	AB + CS group (*n* = 39)	*p* value
Age (years ± SD)	43.6 ± 8.1	46.5 ± 8.5	45.5 ± 6.0	0.232^a^
Gender (M/F)	12/28	10/30	14/25	0.573^b^
Laterality (R/L)	30/10	24/16	24/15	0.300^b^
Dominant side involvement (D/ND)	30/10	24/16	23/16	0.246^b^
PRTEE (points ± SD)	69.12 ± 9.7	66.02 ± 12.8	66.4 ± 11.2	0.440^c^

*SD* Standard deviation

^a^ANOVA

^b^Chi-square

^c^Kruskal–Wallis test

By day 15, all groups had demonstrated a significant improvement in PRTEE scores compared to the baseline levels. When comparing the treatment groups, CS showed a more pronounced reduction than ABI and combined injections (*p* = 0.001). However, there was no statistically significant difference between the ABI and combined groups (*p* = 0.940). By day 30, although the ABI and combined-injection groups showed a significant improvement in PRTEE scores, the functional scores in the CS group had deteriorated. Similar PRTEE scores were obtained in all groups (*p* = 0.096). At day 90, the improvement in the ABI and combined groups continued, but the CS groups showed further deterioration. The combined injection was a more effective treatment compared to ABI or CS alone (Table 2).

**Table 2:** Summary of outcome measures at each assessment point and comparisons both between and within the treatment groups

Variable	Group	Day 0	Day 15	Day 30	Day 90	*p* value
PRTEE (mean ± SD)	ABI	69.1 ± 9.7	53.3 ± 15.0	39.3 ± 15.7	25.5 ± 13.1	0.001^a^
	CS	66.0 ± 12.8	25.9 ± 16.9	31.4 ± 15.5	40.2 ± 15.5	0.001^a^
	ABI + CS	66.4 ± 11.2	52.1 ± 16.4	36.5 ± 19.2	16.9 ± 10.8	0.001^a^
*p* value		0.440^b^	0.001^b^	0.096^b^	0.001^b^	
ABI vs. CS			0.001^c^		0.001^c^	
ABI vs. ABI + CS			0.940^c^		0.015^c^	
CS vs. ABI + CS			0.001^c^		0.001^c^	

The *p* values in the rightmost column refer to comparisons of repeated measurements within the same group, while the row of *p* values refer to comparisons between groups and subgroups

*SD* standard deviation

^a^Friedman two-way ANOVA

^b^Kruskal–Wallis test

^c^Tukey’s test

Analyzing the clinical significance, those who showed a PRTEE score change of less than 37% were labeled as having failed treatment. In contrast, a change of greater than 37% indicated a clinically significant improvement. The combined AB + CS treatment was the most effective, with only 2.6% (*n* = 1) of the patients experienced a failed treatment, while a resounding 97.4% (*n* = 38) achieved a clinically significant improvement. For AB, 15% (*n* = 6) underwent a failed treatment, and 85% (*n* = 34) showed significant improvement. The CS group was the worst group, with 45% (*n* = 18) and 55% (*n* = 22) undergoing a failed treatment and showing significant improvement, respectively (Table 3).

**Table 3:** Comparison of groups according to the minimum clinically important difference in PRTEE score

Treatment group	PRTEE change < 37% (failed treatment)	PRTEE change > 37% (clinically significant ımprovement)	*p* value
AB (*n*, % within group)	6 (15)	34 (85%)	0.001^a^
CS (*n*, % within group)	18 (45)	22 (55%)	
AB + CS (*n*, % within group)	1 (2.6)	38 (97.4%)	
AB vs. CS			0.003
AB + CS vs. CS			0.001
AB vs. AB + CS			0.051

*SD* standard deviation

^a^Chi-square test

The HGS follow-up showed results that paralleled the functional results. By day 15, an improvement in HGS was evident across the groups. The AB injection group registered a 6.7 ± 13.7% rise, the CS group showed the largest increase of 24.1 ± 27.8%, and the AB + CS group reported growth of 12.4 ± 17.9%. Progress continued until day 90, with the AB and AB + CS groups showing marked enhancements in HGS of 29.1 ± 26.9% and 36.6 ± 38.0%, respectively. In contrast, the CS group experienced a slight enhancement to 20.6 ± 27.5%. Notably, intra-group HGS advancements over the evaluation period were statistically significant for both the AB and AB + CS groups (both *p* = 0.001) but not for the CS group (*p* = 0.343) (Table 4).

**Table 4:** Summary of outcome measures at each assessment point and comparisons both between and within the treatment groups

Variable	Group	Day 15	Day 30	Day 90	*p* value
HGS (% change ± SD)	AB	6.7 ± 13.7	18.8 ± 21.3	29.1 ± 26.9	0.001^a^
	CS	24.1 ± 27.8	18.8 ± 22.8	20.6 ± 27.5	0.343^a^
	AB + CS	12.4 ± 17.9	24.2 ± 25.0	36.6 ± 38.0	0.001^a^
*p* value		0.005^b^	0.344^b^	0.036^b^	
AB vs. CS		0.001^c^		0.001^c^	
AB vs. AB + CS		0.447^c^		0.027^c^	
CS vs. AB + CS		0.001^c^		0.001^c^	

The *p* values in the rightmost column refer to comparisons of repeated measurements within the same group, while the row of *p* values refer to comparisons between groups and subgroups

*SD* standard deviation

^a^Friedman two-way ANOVA

^b^Kruskal–Wallis test

^c^Tukey’s test

## Discussion

This randomized clinical trial aimed to examine and compare the efficacies of AB, CS, and a combination of both (AB + CS) for treating lateral epicondylitis (LE). The hypothesis posited that the combined injection might offer superior results in terms of fast symptom resolution and a lower recurrence rate by leveraging the initial action of CS and the regenerative potential of ABI. Our results highlighted that by day 15, all treatment modalities had produced significant improvements in PRTEE scores, with the CS group showing a more pronounced reduction compared to the other groups. However, this early gain in the CS group started to deteriorate by day 30 and continued till day 90, emphasizing the transient relief provided by CS injections. On the other hand, the ABI and combined-injection groups showed continued improvements in PRTEE scores, substantiating the role of these treatments in consistent symptom reduction and potentially addressing intrinsic tendon degeneration. The combined AB + CS treatment emerged as the most effective modality, with substantial clinical improvement and minimal failed treatment instances. This observation implies that a potential synergistic mechanism underlies the combined treatment approach, suggesting that a multimodal therapeutic strategy may yield more enduring and reliable relief for individuals afflicted with lateral epicondylitis.

CS injections have been used since the 1950s as a treatment strategy for LE [3, 16]. Although the mechanism of action could not be explained in detail, it is proposed that CS may decrease some inflammatory mediators and neuropeptides such as Substance P, calcitonin gene-related peptide, and neurokinin-1, which are responsible for the pain. Thus, the injection of CS relieves pain immediately. However, it cannot reverse the degeneration process to regeneration within the tendon, and the symptoms turn back on a few weeks after the injection, when its inhibitory effect ceases. Several authors have shown the temporary effect of CS. Furthermore, some authors even hypothesize that CS injections worsen long-term results by either weakening the tendon or by allowing patients to further aggravate their tendinosis initially by relieving pain in the short term [17]. Similarly, in our study, we observed this pattern clearly.

Edwards and Calandruccio first described AB injection for the treatment of LE. They reported a 79% success rate (22 out of 28 patients) in their series [9]. The mechanism of action of AB injection is also not known, but it is believed that cellular and humoral mediators found in blood initiate the healing cascade of the tendon and reverse the degeneration to regeneration, in contrast to the action of CS. Since then, several authors have shown that AB injection is an effective treatment for LE, with no serious side effects. In the current literature, few studies compare CS and AB injections for the treatment of LE (Table 5) [10, 11, 18–25]. Of these studies, only one study could not demonstrate any significant difference between AB and CS. Wolf et al. performed a randomized clinical trial (RCT) of 28 patients in which AB, CS, and a saline injection were compared. In this study, the patients were evaluated with VAS, DASH, and the patient-related forearm evaluation 2 weeks, 2 months, and 6 months after injection. According to the study’s outcomes, all groups demonstrated an improvement from baseline, but there were no significant differences in any of the groups [18]. Apart from that study, the other studies reported superior clinical outcomes with AB injections. However, the current study introduces a novel perspective by incorporating a combined-treatment approach. The impressive clinical improvement and sustained benefits observed with AB + CS suggest that this combined modality may offer a balanced and superior alternative, providing both immediate relief and long-term recovery. This aligns with the findings of Lee et al., where a combined approach showed better results during the initial period, although the improvements were similar by the 6th month [25].

**Table 5:** Summary of previous studies that compared ABI versus CS injections for the treatment of LE in the current literature

Author	Year	Study design	Patients	Treatment groups	Outcome measures	Follow-up (months)	Conclusion
Kazemi et al.	2010	Single-blinded RCT	60 (30/30)	ABI vs. CS	VAS Nirschl staging QDASH Grip strength Algometry	2	AB is better than CS at 8 weeks
Ozturan et al.	2010	Non-blinded RCT	57 (18/20/19)	ABI vs. CS vs. ESWT	VAS provocative test Functional scale	12	83.3% recovery in ABI group 89.9% recovery in ESWT group 50% recovery in steroid group
Wolf et al.	2011	Single-blinded RCT	28 (10/9/9)	ABI vs. CS vs. Placebo	VAS DASH PRFE	6	No differences between groups in all outcome measures at 6 months
Dojobe CM	2012	Non-blinded RCT	60 (30/30)	ABI vs. CS	VAS Nirschl staging	6	ABI is better than steroid (90% vs. 47% recovery)
Jindal et al.	2013	Single-blinded RCT	50 (25/25)	ABI vs. CS	VAS Nirschl staging	1.5	ABI is better than CS
Singh et al.	2013	Single-blinded RCT	60 (30/30)	ABI vs. CS	PRTEE	3	ABI is better than CS
Arık et al.	2014	Non-blinded RCT	80 (40/40)	ABI vs. CS	VAS PRTEE Grip strength	6	95% recovery in ABI group 62.5% recovery in CS group
Branson et al.	2017	Single-blinded RCT	44 (14/16/14)	ABI vs. CS vs. polidocanol	PRTEE Grip strength	4.5	ABI and polidocanol are better than CS
Kaya et al.	2022	Single-blinded RCT	120 (40/40/40/40)	ABI vs. CS. vs. dextrose vs. splint	VAS PRTEE Grip strength	6	ABI, CS, and dextrose are similar and better than splint
Lee et al.	2022	Single-blinded RCT	129 (69/60)	ABI + dextrose + CS vs. ABI + dextrose	VAS Grip strength	6	ABI + dextrose + CS was better during the first 3 months but similar in the 6th month
Current study	2023	Non-blinded RCT	119 (40/40/39)	ABI vs. CS vs. ABI + CS	PRTEE Grip strength	3	ABI and ABI + CS are better than CS alone; ABI + CS has a fast pain decrease response

*VAS* visual analog scale, *ABI* autologous whole blood Injection, *CS* corticosteroid, *RCT* randomized clinical trial, *PRTEE* patient-rated tennis elbow evaluation, *PRFE* patient-rated forearm evaluation, *ESWT* extracorporal shock wave therapy

*QDASH* The Quick Disabilities of the Arm, Shoulder, and Hand

An ideal treatment of LE should ensure immediate pain relief, a fast return to daily activities and work, and a low recurrence rate. Any treatment method that provides these goals faster than other options is advantageous. In this study, we attempted to optimize the current injection technique by combining two available options. We utilized the immediate pain-relieving effect of CS and the long-term regenerative effect of AB. Although AB injection alone and AB plus CS injection resulted in similar final clinical outcomes at the final follow-up, the the combined treatment had longer-lasting effects on function.

This study has some strengths and limitations. The follow-up period was relatively short, limiting the assessment of the long-term efficacies and recurrence rates of the treatment modalities. This study was not blinded, which could have introduced bias into the results. On the other hand, this was a randomized clinical trial, which provides a high level of evidence. No patients were lost during the study period, and all patients had similar initial characteristics regarding several parameters.

## Conclusions

While AB and CS individually offer distinct benefits, a combined AB + CS approach optimizes therapeutic outcomes, providing swift and sustained functional improvement with a lower recurrence rate. These findings have substantial clinical implications, suggesting a balanced, multimodal treatment strategy for the enhanced recovery of patients with LE. Among current publications, this study stands as a pioneering effort to merge ABI and CS into a treatment for LE. Utilizing both CS and AB injections appears to be a promising method, as it blends the immediate pain alleviation provided by CS with ABI’s sustained restorative effects. Notably, this approach is cost-effective, straightforward, and devoid of known complications. In the future, other combinations of injections or therapeutic methods may be explored.

## Data Availability

The datasets used and/or analyzed during the current study are available from the corresponding author upon reasonable request.

## Abbreviations

LE: Lateral epicondylitis
PRTEE: Patient-rated tennis elbow evaluation
R: Right
L: Left
D: Dominant
ND: Non-dominant
AB: Autologous blood
ABI: Autologous blood injection
CS: Corticosteroid
HGS: Hand grip strength
